# Identification of loss-of-function RyR2 mutations associated with idiopathic ventricular fibrillation and sudden death

**DOI:** 10.1042/BSR20210209

**Published:** 2021-04-22

**Authors:** Xiaowei Zhong, Wenting Guo, Jinhong Wei, Yijun Tang, Yingjie Liu, Joe Z. Zhang, Vern Hsen Tan, Lin Zhang, Ruiwu Wang, Peter P. Jones, Carlo Napolitano, Silvia G. Priori, S.R. Wayne Chen

**Affiliations:** 1 The Libin Cardiovascular Institute, Department of Physiology and Pharmacology, University of Calgary, Calgary, AB, Canada; 2 Department of Physiology and HeartOtago, University of Otago, Dunedin, New Zealand; 3 Foothills Hospital, the Libin Cardiovascular Institute, University of Calgary, Calgary, AB, Canada; 4 Department of Cardiology, Changi General Hospital, Singapore; 5 Division of Cardiology and Molecular Cardiology, IRCCS Maugeri Foundation-University of Pavia, Pavia, Italy; 6 Department of Molecular Medicine, University of Pavia, Pavia, Italy; 7 Molecular Cardiology Laboratory, Centro de Investigaciones Cardiovasculares Carlos III, Madrid, Spain

**Keywords:** Disease mutations, Ryanodine receptor, Sarcoplasmic reticulum, Sudden cardiac death, Ventricular fibrillation, Ventricular tachyarrhythmias

## Abstract

Mutations in cardiac ryanodine receptor (RyR2) are linked to catecholaminergic polymorphic ventricular tachycardia (CPVT). Most CPVT RyR2 mutations characterized are gain-of-function (GOF), indicating enhanced RyR2 function as a major cause of CPVT. Loss-of-function (LOF) RyR2 mutations have also been identified and are linked to a distinct entity of cardiac arrhythmia termed RyR2 Ca^2+^ release deficiency syndrome (CRDS). Exercise stress testing (EST) is routinely used to diagnose CPVT, but it is ineffective for CRDS. There is currently no effective diagnostic tool for CRDS in humans. An alternative strategy to assess the risk for CRDS is to directly determine the functional impact of the associated RyR2 mutations. To this end, we have functionally screened 18 RyR2 mutations that are associated with idiopathic ventricular fibrillation (IVF) or sudden death. We found two additional RyR2 LOF mutations E4146K and G4935R. The E4146K mutation markedly suppressed caffeine activation of RyR2 and abolished store overload induced Ca^2+^ release (SOICR) in human embryonic kidney 293 (HEK293) cells. E4146K also severely reduced cytosolic Ca^2+^ activation and abolished luminal Ca^2+^ activation of single RyR2 channels. The G4935R mutation completely abolished caffeine activation of and [^3^H]ryanodine binding to RyR2. Co-expression studies showed that the G4935R mutation exerted dominant negative impact on the RyR2 wildtype (WT) channel. Interestingly, the RyR2-G4935R mutant carrier had a negative EST, and the E4146K carrier had a family history of sudden death during sleep, which are different from phenotypes of typical CPVT. Thus, our data further support the link between RyR2 LOF and a new entity of cardiac arrhythmias distinct from CPVT.

## Introduction

The cardiac ryanodine receptor (RyR2) is a sarcoplasmic reticulum (SR) Ca^2+^ release channel essential for excitation–contraction coupling in the heart [1,2]. RyR2 is also a critical player in the pathogenesis of various cardiac arrhythmias and cardiomyopathies [2–5]. Naturally occurring mutations in RyR2 have frequently been associated with catecholaminergic polymorphic ventricular tachycardia (CPVT), but also with other phenotypes such as idiopathic ventricular fibrillation (IVF), atrial fibrillation (AF), and cardiomyopathies [6–19]. An unresolved important question is how mutations in the same *RYR2* gene can cause such a wide spectrum of cardiac disorders.

A major effort towards addressing this question has been to understand the functional impact of disease-causing RyR2 mutations. To date, many RyR2 mutations have been characterized at the molecular, cellular, or whole animal levels [3–5]. Interestingly, nearly all RyR2 mutations that have been characterized enhance the response of the RyR2 channel to stimuli (i.e. gain-of-function, GOF). For instance, single-channel studies demonstrated that many CPVT-associated RyR2 mutations enhance the sensitivity of the RyR2 channel to luminal Ca^2+^ activation [20,21]. Some CPVT RyR2 mutations have also been shown to increase the sensitivity of the channel to activation by luminal and/or cytosolic Ca^2+^ [20,22–25]. At the cellular level, CPVT RyR2 mutations enhance spontaneous Ca^2+^ release in the form of Ca^2+^ sparks, Ca^2+^ oscillations, or Ca^2+^ waves under conditions of SR Ca^2+^ overload [20–22,24,26–30]. This store overload induced Ca^2+^ release (SOICR) can lead to delayed afterdepolarizations (DADs), triggered activities, and ventricular tachyarrhythmias [5,31–33]. RyR2 mutations associated with dilated cardiomyopathy affect the termination of SR Ca^2+^ release by reducing the termination threshold, leading to prolonged SR Ca^2+^ release [34]. Furthermore, RyR2 mutations located in the central domain increase the sensitivity of the RyR2 channel to activation by cytosolic Ca^2+^ [35,36]. Interestingly, some of these central domain RyR2 mutations are associated with AFs [17,19]. Recent three-dimensional structural studies revealed that a large number of disease-causing RyR2 mutations are clustered at domain interfaces [37–44]. These mutations are thought to weaken domain–domain interactions that are important for stabilizing the closed state of the channel, thus facilitating channel opening and spontaneous SR Ca^2+^ release [45]. Overall, these observations have led to a general belief that disease-linked RyR2 mutations cause GOF defects, leading to inappropriate activation of the channel and excessive SR Ca^2+^ release that can precipitate into cardiac arrhythmias, cardiomyopathies, or sudden death [5].

In addition to disease-associated RyR2 GOF mutations, we have recently shown that RyR2 loss-of-function (LOF) mutations are causative for a novel inherited cardiac arrhythmia syndrome that we have termed RyR2 Ca^2+^ release deficiency syndrome (CRDS) [46]. Importantly, the phenotypes of the RyR2-CRDS are distinct from those of CPVT. Unlike CPVT, RyR2-CRDS lacks the catecholamine-induced ventricular ectopy [46]. The arrhythmogenic mechanism of RyR2-CRDS is also different from that of CPVT. Animal studies suggest that VTs in RyR2-CRDS arise secondary to substantial electrophysiological remodeling that increases the susceptibility to ventricular arrhythmias via early-afterdepolarization (EAD)-mediated re-entrant mechanism [46]. Because of these differences, the diagnosis, management, and treatment of RyR2-CRDS would be different from those of CPVT. However, although RyR2-CRDS is a life-threatening arrhythmogenic disorder distinct from CPVT, there are currently no diagnostic tests for the disorder in humans. The standard exercise-stress test for CPVT is unable to distinguish RyR2-CRDS from normal individuals.

In light of the link between RyR2 LOF and RyR2-CRDS, another approach to assess the risk for RyR2-CRDS is to screen for RyR2 mutations and functionally characterize their impact. To date, there are ten RyR2 LOF mutations (Q3774L, I3995V, D4112N, T4196I, K4594R/I2075T, D4646A, I4855M, A4860G, Q4879H, and S4938F) that have been reported [46]. There are certainly more RyR2 LOF mutations and identifying these LOF RyR2 mutations is critical to prevent sudden death. To this end, in the present study, we determined the functional impact of a large number of known RyR2 mutations that are associated with IVF or sudden unexplained death (SUD). Our site-directed mutagenesis studies and functional screening led to the identification of 2 additional LOF RyR2 mutations (E4146K and G4935R) associated with IVF and sudden death. Thus, ventricular arrhythmias associated with RyR2 LOF represents a significant portion of RyR2-linked arrhythmogenic disorders that are yet to be explored.

## Materials and methods

### Materials

Human embryonic kidney 293 (HEK293) cell line, plasmid pcDNA3, plasmid pcDNA5, Tris-HCl (pH 8.8), MgSO_4_, Triton X-100, bovine serum albumin (BSA), dATP, dCTP, dGTP and dTTP (Amersham), DNA polymerase (Stratagene), QIA quick PCR Purification Kit, Flp-In T-Rex Core Kit (Invitrogen), phosphate-buffered saline (PBS) (137 mM NaCl, 8 mM Na_2_HPO_4_, 1.5 mM KH_2_PO_4_ and 2.7 mM KCl), hygromycin (Invitrogen), Dulbecco’s Modified Eagle’s Medium (DMEM), fetal bovine serum, HEPES buffer (274 mM NaCl, 1.8 mM Na_2_HPO_4_ and 50 mM HEPES, pH 7.04), KRH (Krebs–Ringer–HEPES) buffer (125 mM NaCl, 5 mM KCl, 1.2 mM KH_2_PO_4_, 6 mM glucose, 1.2 mM MgCl_2_ and 25 mM HEPES, pH 7.4), CaCl_2_, fluo 3, sulfinpyrazone, Fura-2 acetoxymethyl ester (Fura-2 AM), tetracycline, pluronic F-127, caffeine, EGTA, EDTA, Tris, CHAPS, soybean phosphatidylcholine, DTT, benzamidine, leupeptin, pepstatin A, aprotinin, PMSF, L-glutamine, penicillin, nonessential amino acids, Tween-20, skimmed-milk powder, anti-RyR antibody (34c), anti-mouse IgG (H&L) antibodies, enhanced chemiluminescence kit (Pierce), [^3^H]ryanodine (PerkinElmer), ryanodine (Abcam).

## Methods

### Construction of RyR2 mutations

All RyR2 point mutations were generated by using the overlap extension PCR method as described previously [47,48]. The PCRs were carried out in a 100-μl reaction buffer containing 20 mM Tris-HCl (pH 8.8), 10 mM KCl, 2.0 mM MgSO_4_, 0.1% Triton X-100, 0.1 mg/ml BSA, 50 ng of each DNA primer, 200 μM each of dATP, dCTP, dGTP and dTTP (Amersham), 1 unit of pfu DNA polymerase (Stratagene), and 100 ng of template cDNA. All of the PCR products were purified using the QIA quick PCR Purification Kit. cDNA fragments containing the desired mutations were removed from the PCR products and used to replace the corresponding wildtype (WT) fragments in the full-length mouse RyR2 cDNA in the expression plasmid pcDNA5 [49]. The mutations and sequence of the PCR products were confirmed by DNA sequencing.

### Generation of stable, inducible HEK293 cell lines expressing RyR2 WT or mutants

Stable, inducible HEK293 cell lines expressing RyR2 WT or the E4146K mutant were generated using the Flp-In T-REx Core Kit from Invitrogen. Briefly, Flp-In T-REx-293 cells were co-transfected with the inducible expression vector pcDNA5/FRT/TO containing the mutant cDNAs and the pOG44 vector encoding the Flp recombinase in 1:5 ratios using the Ca^2+^ phosphate precipitation method. The transfected cells were washed with PBS (137 mM NaCl, 8 mM Na_2_HPO_4_, 1.5 mM KH_2_PO_4_, 2.7 mM KCl) 24 h after transfection followed by a change into fresh media for 24 h. The cells were then washed again with PBS, harvested, and plated on to new dishes. After the cells had attached (∼4 h), the growth medium was replaced with a selection medium containing 200 μg/ml hygromycin (Invitrogen). The selection medium was changed every 3–4 days until the desired number of cells was grown. The hygromycin-resistant cells were pooled, aliquoted, and stored at −80°C. These positive cells are believed to be isogenic, because the integration of RyR2 cDNA is mediated by the Flp recombinase at a single FRT site.

### Caffeine-induced Ca^2+^ release measurements

Free cytosolic Ca^2+^ concentration in transfected HEK293 cells was measured using the fluorescence Ca^2+^ indicator dye fluo-3-AM as described previously [49]. HEK293 cells grown on 100-mm tissue culture dishes for 18–20 h after subculture were transfected with 12–16 µg of WT or mutant RyR2 cDNA. Cells grown for 18–20 h after transfection were washed four times with PBS and incubated in KRH buffer without MgCl_2_ and CaCl_2_ (KRH buffer: 125 mM NaCl, 5 mM KCl, 1.2 mM KH_2_PO_4_, 6 mM glucose, 1.2 mM MgCl_2_, 2 mM CaCl_2_, and 25 mM HEPES, pH 7.4) at room temperature for 40 min, and at 37°C for 40 min. After being detached from culture dishes, cells were collected by centrifugation at 1000 rpm for 3 min in a Beckman TH-4 rotor. Cell pellets were washed twice with KRH buffer and loaded with 10 µM fluo 3 in DMEM at room temperature for 60 min, followed by washing with KRH buffer three times, and resuspended in 150 µl KRH buffer plus 0.1 mg/ml BSA and 250 µM sulfinpyrazone. The fluo 3-loaded cells were added to 2 ml (final volume) KRH buffer in a cuvette. Fluorescence intensity of fluo 3 at 530 nm was measured before and after repeated additions or single additions of various concentrations of caffeine in an SLM-Aminco series 2 luminescence spectrometer with 480 nm excitation at 25°C (SLM Instruments, Urbana, IL). The peak level of each caffeine-induced Ca^2+^ release was determined and normalized to the highest level (100%) of caffeine-induced Ca^2+^ release for each experiment. The normalized data were fitted with the Hill equation.

### Single-cell cytosolic Ca^2+^ imaging of HEK293 cells

Cytosolic Ca^2+^ levels in stable, inducible HEK293 cells expressing RyR2 WT or the E4146K mutant channels were monitored using single-cell Ca^2+^ imaging and the fluorescent Ca^2+^ indicator dye Fura-2 AM as described previously [20,21]. Briefly, cells grown on glass coverslips for 18–22 h after induction by 1 μg/ml tetracycline were loaded with 5 μM Fura-2 AM in KRH buffer (125 mM NaCl, 5 mM KCl, 1.2 mM KH_2_PO_4_, 6 mM glucose, 1.2 mM MgCl_2_ and 25 mM HEPES, pH 7.4) plus 0.02% pluronic F-127 and 0.1 mg/ml BSA for 20 min at room temperature (23°C). The coverslips were then mounted in a perfusion chamber (Warner Instruments, Hamden, CT, U.S.A.) on an inverted microscope (Nikon TE2000- S). The cells were continuously perfused with KRH buffer containing increasing extracellular Ca^2+^ concentrations (0, 0.1, 0.2, 0.3, 0.5, 1.0, and 2.0 mM). Caffeine (10 mM) was applied at the end of each experiment to confirm the expression of active RyR2 channels. Time-lapse images (0.25 frame/s) were captured and analyzed with the Compix Simple PCI 6 software (Compix Inc., Sewickley, PA, U.S.A.). Fluorescence intensities were measured from regions of interest centered on individual cells. Only cells that responded to caffeine were analyzed. The filters used for Fura-2 imaging are exciters: 340 ± 26 and 387 ± 11 nm, and emitter: 510 ± 84 nm with a dichroic mirror (410 nM).

### Single-channel recordings in planar lipid bilayers

Recombinant RyR2 WT and mutant channels were purified from cell lysate prepared from HEK293 cells transfected with RyR2 WT or the E4146K mutant cDNAs by sucrose density gradient centrifugation as described previously [50]. Bilayers were formed across a 250-µm hole in a Delrin partition separating two chambers. The trans chamber (800 µl) was connected to the head stage input of an Axopatch 200A amplifier (Axon Instruments, Austin, TX). The *cis* chamber (1.2 ml) was held at virtual ground. A symmetrical solution containing 250 mM KCl and 25 mM HEPES (pH 7.4) was used for all recordings, unless indicated otherwise. A 4-µl aliquot (≈1 µg of protein) of the sucrose density gradient-purified recombinant RyR2 WT or E4146K mutant channels was added to the *cis* chamber. Spontaneous channel activity was always tested for sensitivity to EGTA and Ca^2+^. The chamber to which the addition of EGTA inhibited the activity of the incorporated channel presumably corresponds to the cytosolic side of the Ca^2+^ release channel. The direction of single channel currents was always measured from the luminal to the cytosolic side of the channel, unless mentioned otherwise. Recordings were filtered at 2500 Hz. Data analyses were carried out using the pclamp 8.1 software package (Axon Instruments). Note that in our single channel studies, we used a wide range of Ca^2+^ concentrations from 45 nM to 40 mM. it is challenging to prepare the exact Ca^2+^ concentrations in the nanomolar and submicromolar range confirmed with a Ca^2+^ electrode. Hence, we estimated the free Ca^2+^ concentrations using the computer program of Fabiato and Fabiato [51]. To minimize potential impact of variable estimations of free Ca^2+^ concentrations, we always use the same Ca^2+^ stock solutions to test the Ca^2+^ response of the WT and mutant channels under the same conditions.

### Western blotting

HEK293 cells transfected with RyR2 WT or the RyR2-E4146K mutant cDNAs were washed with PBS plus 2.5 mM EDTA and harvested in the same solution by centrifugation at 2000 rpm for 10 min in a Beckman TH-4 rotor. The cells were then washed with PBS without EDTA and centrifuged again at 2000 rpm for 10 min. The PBS washed cells were solubilized in a lysis buffer containing 25 mM Tris, 50 mM HEPES (pH 7.4), 137 mM NaCl, 1% CHAPS, 0.5% soybean phosphatidylcholine, 2.5 mM DTT, and a protease inhibitor mix (1 mM benzamidine, 2 µg/ml leupeptin, 2 µg/ml pepstatin A, 2 µg/ml aprotinin, 0.5 mM PMSF). This mixture was incubated on ice for 1 h. Cell lysate was obtained by centrifuging twice at 16000×***g*** in a microcentrifuge at 4°C for 30 min to remove unsolubilized materials. The RyR2 WT and mutant proteins were subjected to 6% SDS/PAGE [52] and transferred to nitrocellulose membranes at 45 V for 18–20 h at 4°C in the presence of 0.01% SDS [53]. The nitrocellulose membranes containing the transferred proteins were blocked for 30 min with PBS containing 0.5% Tween-20 and 5% skimmed-milk powder. The blocked membrane was incubated with the anti-RyR antibody (34c) (1:1000) and then incubated with the secondary anti-mouse IgG (H&L) antibodies conjugated to horseradish peroxidase (1:20000). After washing for 5 min, three times, the bound antibodies were detected using an enhanced chemiluminescence kit from Pierce.

### [^3^H]Ryanodine binding assay

Equilibrium [^3^H]ryanodine binding to cell lysates was performed as described previously [49,54] with some modifications. Cell lysates were incubated with 5 nM [^3^H]ryanodine at 37°C for 2 h in 300 µl of a binding solution containing 500 mM KCl, 25 mM Tris, 50 mM HEPES (pH 7.4). Free [Ca^2+^] (0.1 nM to 100 µM) was adjusted by EGTA and CaCl_2_ solutions using the computer program of Fabiato and Fabiato [51]. At the completion of incubation, samples were diluted with 5 ml of ice-cold washing buffer containing 25 mM Tris (pH 8.0) and 250 mM KCl, and filtered through Whatman GF/B filters presoaked with 1% polyethylenimine. Filters were washed immediately with 2 × 5 ml of the same buffer. The amount of [^3^H]ryanodine retained in filters was determined by liquid scintillation counting. Specifically bound [^3^H]ryanodine was calculated by subtracting nonspecific binding that was determined in the presence of 50 µM unlabeled ryanodine. All binding assays were performed in duplicate. [^3^H]ryanodine binding data were fitted with the Hill equation using the Prism 8 (GraphPad Software, San Diego, California, U.S.A.).

### Statistical analysis

All values shown are mean ± SEM unless indicated otherwise. To test for differences between groups, we used Student’s *t* test (two-tailed) or one-way ANOVA with post hoc tests. A *P*-value <0.05 was considered to be statistically significant.

### Human studies

Human studies were approved by IRCCS Fondazione Maugeri ethical IRB and in agreement with the Declaration of Helsinki. Patients were referred to the Molecular Cardiology Clinics of the Maugeri Foundation for evaluation of family history of sudden cardiac death (SCD) and underwent clinical evaluation and genetic testing. All patients or their guardians provided written informed consent.

## Results

### Identification of LOF RyR2 mutations associated with sudden death

**Figure 1:**
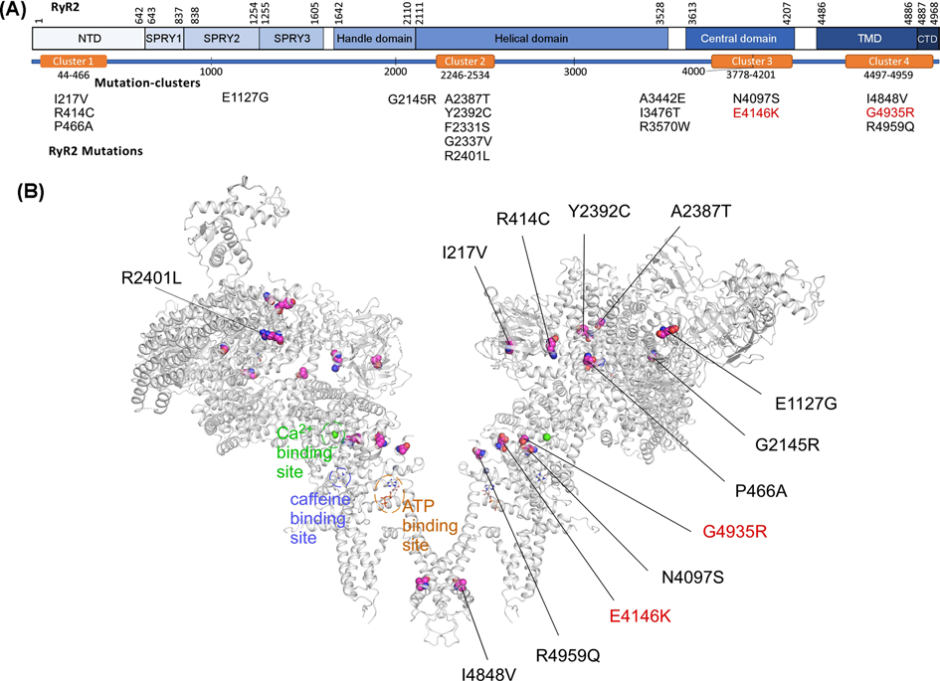
Identification and location of RyR2 mutations associated with IVF and SCD (**A**) A schematic diagram of the linear sequence of RyR2. Major structural domains of RyR2 are depicted as solid blue boxes. The orange boxes indicate four disease-associated mutation clusters (mutation hotspots) in RyR2. IVF- and SCD-associated RyR2 mutations identified were listed underneath their corresponding domains. **(B)** Locations of RyR2 mutations in the three-dimensional structure of RyR2 (6JI0). The three-dimensional locations of RyR2 mutations, F2331S, G2337V, A3442E, I3476T, and R3570W have not been resolved and thus were not shown in the three-dimensional structure. The bindings sites for Ca^2+^, caffeine, and ATP are also shown.

Among hundreds of disease-associated RyR2 mutations, there are only a handful of known LOF RyR2 mutations characterized to date [8,46,55–58]. The impact of most of the disease-associated RyR2 mutations has not been functionally characterized. It has become imperative to identify patients with RyR2 LOF mutations, as their phenotypes are effectively concealed prior to their onset of ventricular arrhythmias or sudden death. To this end, we performed functional screening of a large number of RyR2 mutations associated with IVF and/or SUD (Supplementary data). These include RyR2 mutations: I217V [59], R414C [60], P466A [61], E1127G (new), G2145R [62], F2331S [60], G2337V [63], A2387T [61], Y2392C [13], R2401L [60], A3442E (new), I3476T (new), R3570W [62], N4097S [59], E4146K [59], I4848V [61], G4935R [64], and R4959Q [65]. Of these mutations, E1127G, A3442E, and I3476T are novel. Most of these mutations are located in one of the four disease hotspots in RyR2 (Figure 1). All these mutation carriers were labelled as IVF patients or were associated with SUD. It is unclear whether these mutations are associated with the typical CPVT that is caused by GOF RyR2 mutations or with the newly identified RyR2-CRDS that is caused by LOF RyR2 mutations [46].

**Figure 2:**
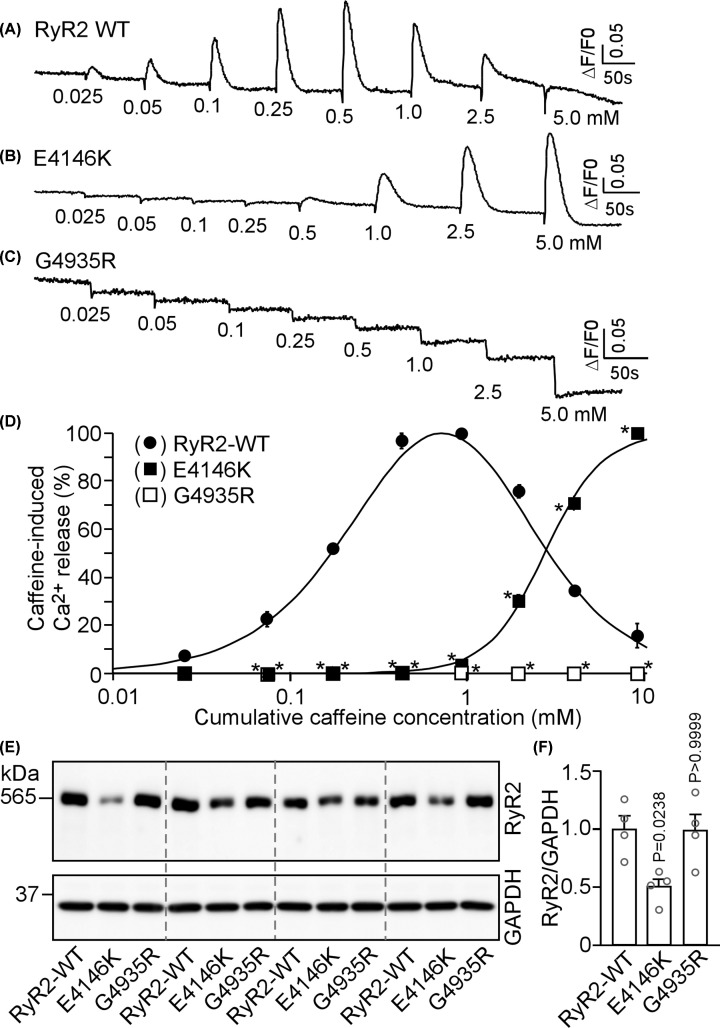
Effects of RyR2 E4146K and G4935R mutations associated with sudden death on caffeine-induced Ca^2+^ release and their expression in HEK293 cells HEK293 cells were transfected with RyR2 WT (**A**), E4146K (**B**), and G4935R (**C**). Fluorescence intensity of the fluo-3-loaded transfected cells was monitored continuously before and after each caffeine addition. The numbers (under the traces) indicate caffeine concentrations. Traces shown are from representative experiments. (**D**) Cumulative caffeine concentration–Ca^2+^ release relationships in HEK293 cells transfected with RyR2 WT, E4146K, and G4935R. Data shown are mean ± SEM (*n*=3). (**E,F**) HEK293 cells were transfected with RyR2 WT, E4146K, or G4935R. Cell lysates were prepared from these transfected cells and used for immunoblotting analysis. The same amount of cell lysate was used for immunoblotting using the anti-RyR2 antibody. Data shown are mean ± SEM (*n*=4, *P <0.05 vs. WT).

**Figure 3:**
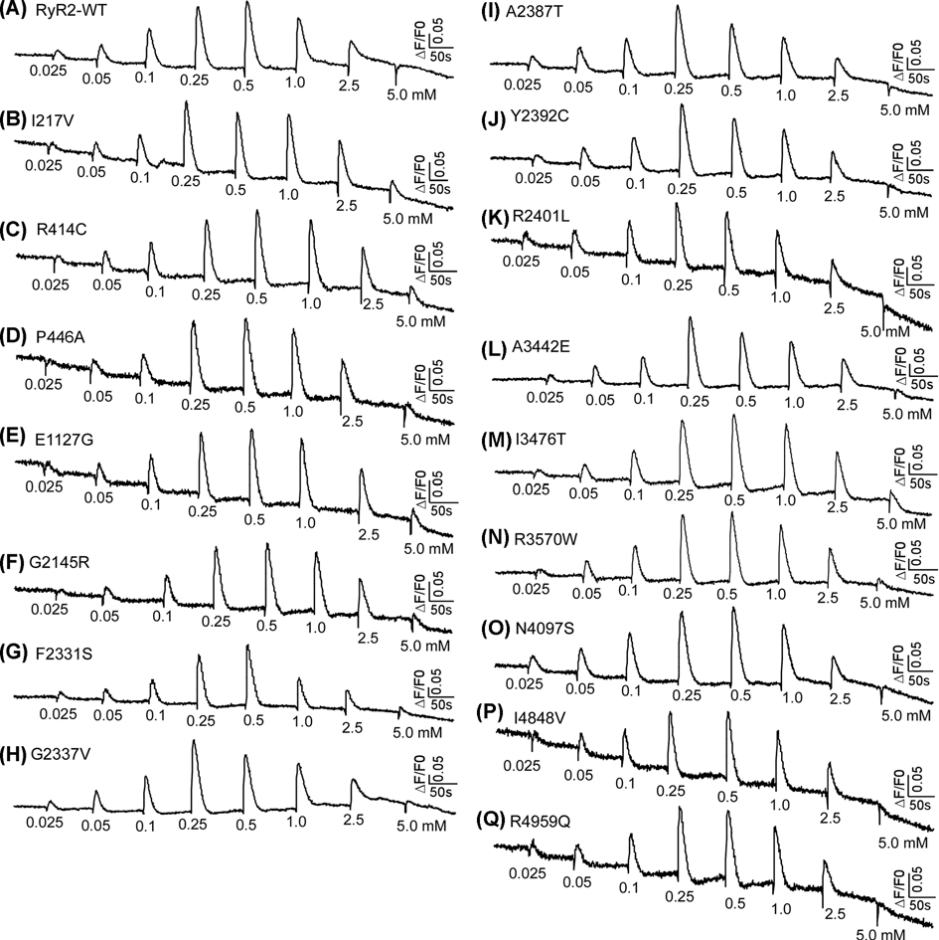
Effect of RyR2 mutations on caffeine-induced Ca^2+^ release in HEK293 cells HEK293 cells were transfected with RyR2 WT or mutants. Fluorescence intensity of the fluo-3-loaded transfected cells was monitored continuously before and after each caffeine addition. The numbers (under the traces) indicate caffeine concentrations. Traces shown are from representative experiments (*n*=3). (**A**) RyR-WT. (**B-Q**) RyR2 mutants tested in the caffeine-induced Ca^2+^ release experiments.

**Figure 4:**
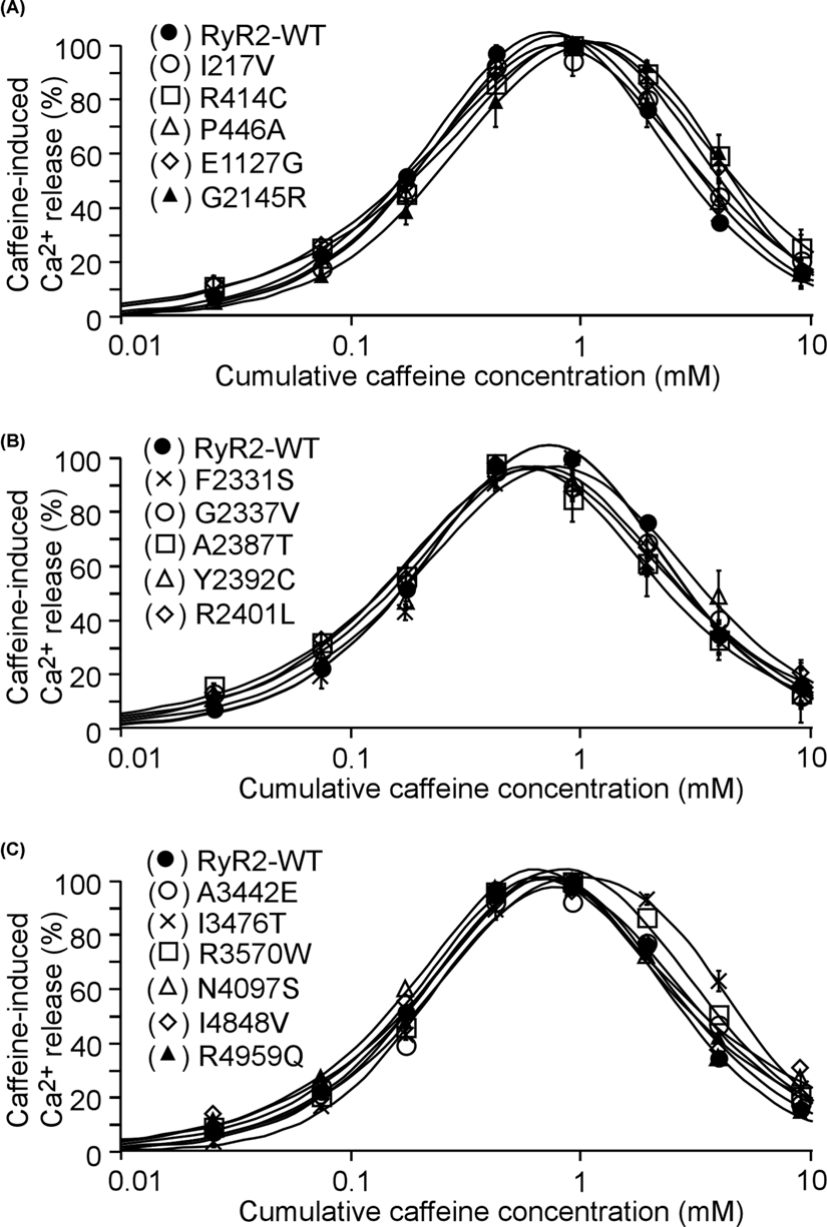
Impact of RyR2 mutations on caffeine-induced Ca^2+^ release in HEK293 cells HEK293 cells were transfected with RyR2 WT or mutants. Fluorescence intensity of the fluo-3-loaded transfected cells was monitored continuously before and after each caffeine addition. (**A–C**) Cumulative caffeine concentration–Ca^2+^ release relationships in HEK293 cells transfected with RyR2 WT or mutants. Note that the caffeine responses of these mutants are similar to that of RyR2 WT. Data shown are mean ± SEM (*n*=3).

To address this question, we assessed the impact of each of the 18 mutations on RyR2 function by measuring caffeine induced Ca^2+^ release in HEK293 cells. Figure 2 shows intracellular Ca^2+^ release induced by sequential additions of increasing concentrations of caffeine in HEK293 cells transfected with the RyR2 WT, the E4146K or G4935R mutants using the fluorescent Ca^2+^ indicator dye fluo-3 AM. HEK293 cells expressing RyR2 WT responded to caffeine with an activation threshold of ∼0.05 mM. The level of Ca^2+^ release increased progressively with each consecutive addition of caffeine from 0.05 mM up to 1.0 mM, and then decreased with further additions of caffeine (2.5 and 5 mM). This decrease is likely due to the depletion of the intracellular Ca^2+^ stores by the prior additions of caffeine (0.025–1.0 mM) (Figure 2A,D). On the other hand, the E4146K mutation markedly suppressed the caffeine response with an activation threshold of ∼1 mM, while the G4935R mutation completely abolished caffeine response (Figure 2B–D). Immunoblotting analysis showed that the expression of the E4146K mutant in HEK293 cells was markedly reduced, whereas the expression of the G4935R mutant was unchanged compared with that of the RyR2 WT (Figure 2E,F, Supplementary Figures S1 and S2). Other RyR2 mutants, including I217V, R414C, P466A, E1127G, G2145R, F2331S, G2337V, A2387T, Y2392C, R2401L, A3442E, I3476T, R3570W, N4097S, I4848V, and R4959Q, exhibited a caffeine response similar to that of the RyR2 WT (Figures 3 and 4). Note that the R414C mutation was previously reported to have no significant impact on the Ca^2+^ dependent activation of [^3^H]ryanodine binding to RyR2 [24]. Therefore, these functional screening identified 2 additional RyR2 mutations (E4146K and G4935R) that substantially suppress or abolish RyR2 function. However, it is important to emphasize that RyR2 mutations that displayed a caffeine response similar to that of RyR2 WT may alter other aspects of channel function. Thus, the pathogenicity of RyR2 mutations with a WT-like caffeine response is unclear and is yet to be determined.

### The RyR2 E4146K mutation abolishes SOICR

**Figure 5:**
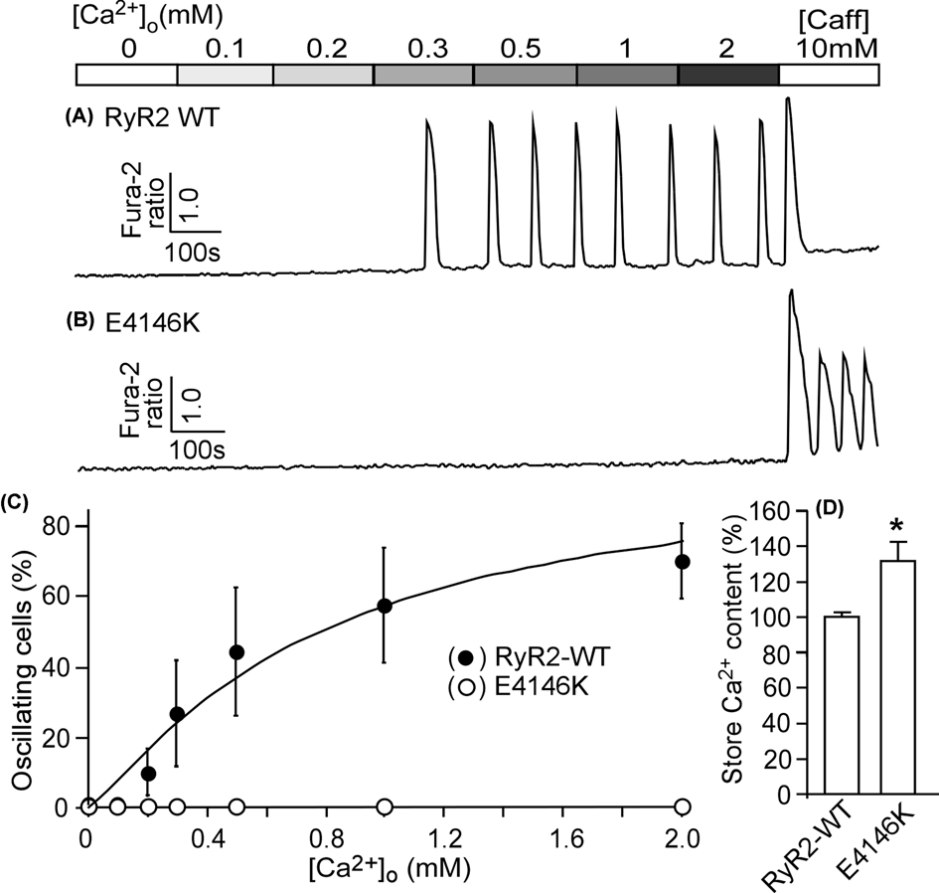
The RyR2-E4146K mutaton abolishes store-overload induced Ca^2+^ release (SOICR) in HEK293 cells Stable, inducible HEK293 cells expressing RyR2 WT or E4146K were loaded with 5 μM Fura-2 AM in KRH buffer. The cells were then perfused continuously with KRH buffer containing increasing levels of extracellular Ca^2+^ (0–2 mM) to induce SOICR. Fura-2 ratios of representative RyR2 WT (**A**) and E4146K (**B**) cells were recorded using single cell Ca^2+^ imaging. (**C**) The percentages of RyR2 WT (691 cells) and E4146K (466) cells that display Ca^2+^ oscillations at various extracellular Ca^2+^ concentrations. Note that no SOICR was detected in HEK293 cells expressing the E4146K mutant. (**D**) ER store Ca^2+^ content in RyR2 WT or E4146K mutant expressing HEK293 cells estimated by measuring the amplitude of caffeine (10 mM) induced Ca^2+^ release. Data shown are mean ± SEM (*n*=3–5, *P<0.05 vs. WT).

We have previously shown that CPVT-linked RyR2 mutations enhance the propensity for SOICR [20,21]. To assess the effect of RyR2 mutation E4146K on SOICR, HEK293 cells expressing RyR2 WT or the E4146K mutation were perfused with elevating extracellular Ca^2+^ (0–2.0 mM) to induce spontaneous Ca^2+^ oscillations as described previously [20,21]. The resultant SOICR was then monitored by using a fluorescence Ca^2+^ indicator, fura-2 AM, and single cell Ca^2+^ imaging. As shown in Figure 5, elevating extracellular Ca^2+^ induced SOICR in HEK293 cells expressing RyR2 WT (Figure 5A,C), whereas, no SOICR was detected in HEK293 cells expressing the RyR2-E4146K mutant (Figure 5B,C), despite the increased ER store Ca^2+^ content compared with that in WT cells (Figure 5D). Thus, opposite to the effect of CPVT-linked RyR2 GOF mutations, the RyR2 E4146K LOF mutation abolishes SOICR in HEK293 cells.

### The E4146K mutation diminishes luminal Ca^2+^ activation of single RyR2 channels

**Figure 6:**
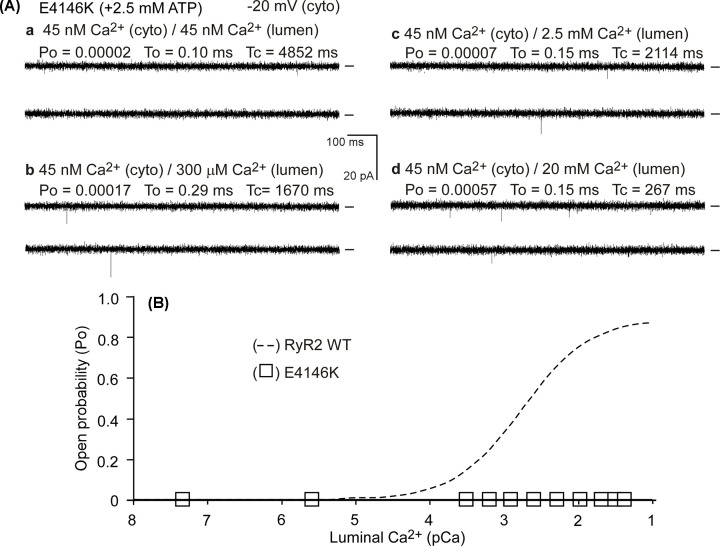
Effect of E4146K on luminal Ca^2+^ activation of single RyR2 channels Single channel activities of the E4146K mutant (**A**) were recorded in a symmetrical recording solution containing 250 mM KCl and 25 mM HEPES (pH 7.4). The Ca^2+^ concentration on both the cytoplasmic and the luminal face of the channel was adjusted to ∼45 nM (panel **a**). The luminal Ca^2+^ concentration was then increased to various levels by an addition of aliquots of CaCl_2_ solution (panels **b**–**d**). Recording potentials were −20 mV. Openings were downward and baselines indicated (short bars). Open probability (Po), mean open time (To), and mean closed time (Tc) are shown. (**B**) The relationships between Po and luminal Ca^2+^ concentrations (pCa) of single E4146K (open squares) mutant channels are shown. Data points shown are mean ± SEM from 7 E4146K single channels. The Po-luminal Ca^2+^ relationship (dashed line) of single RyR2 WT channels was taken from a previous study [46] where E4146K was part of the study.

The impact of the LOF RyR2-E4146K mutation on channel function was further characterized at the single channel level. We first assessed whether the E4146K mutation affects luminal Ca^2+^ activation of RyR2. To this end, we incorporated single RyR2 WT or the E4146K mutant channels into planar lipid bilayers and determined their single channel properties (open probability, Po, mean open time, To, and mean closed time Tc) in the presence of 45nM cytosolic Ca^2+^ and a wide range of luminal Ca^2+^ concentrations (45 nM to 40 mM). As shown in Figure 6, luminal Ca^2+^ up to 40 mM did not activate single E4146K mutant channels, whereas, single RyR2 WT channels were activated by luminal Ca^2+^ under the same conditions (Figure 6A,B). Thus, the E4146K mutation diminishes luminal Ca^2+^ activation of single RyR2 channels.

### The E4146K mutation suppresses cytosolic Ca^2+^ activation of RyR2

**Figure 7:**
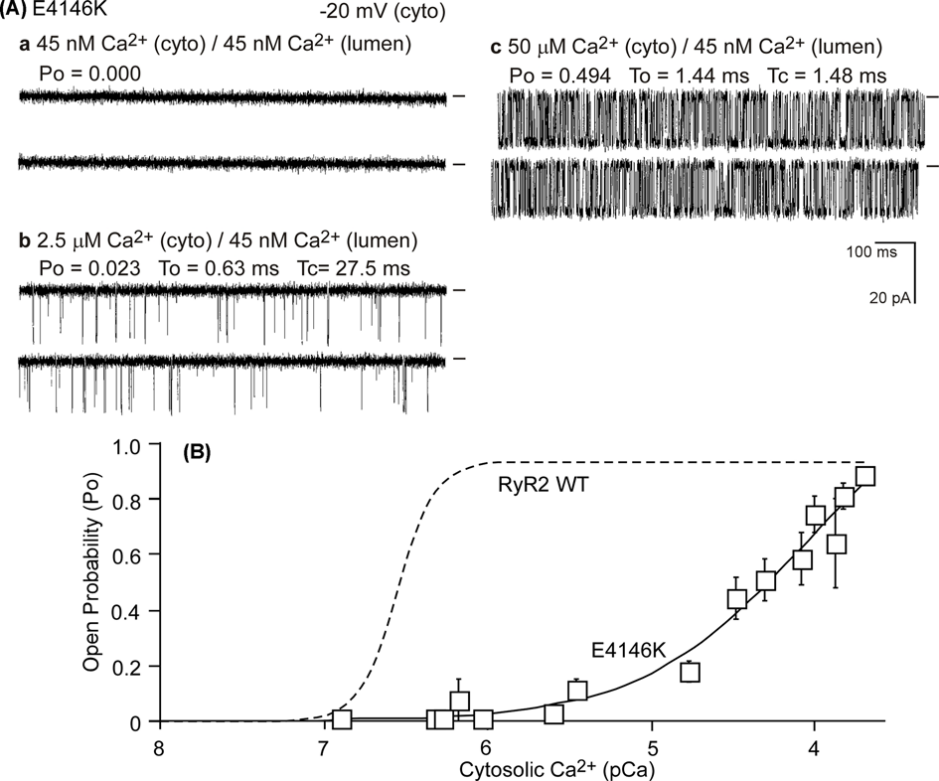
Impact of the E4146K mutation on cytosolic Ca^2+^ activation of single RyR2 channels Single channel activities of the E4146K mutant (**A**) were recorded in a symmetrical recording solution containing 250 mM KCl and 25 mM HEPES (pH 7.4) and in the presence of ∼45 nM luminal Ca^2+^ and various concentrations of cytosolic Ca^2+^ (panels a-c). (**B**) The relationship between Po and cytosolic Ca^2+^ concentrations (pCa) of single E4146K (open squares) mutant channels are shown. Data points shown are mean ± SEM from 14 E4146K single channels. The Po-cytosolic Ca^2+^ relationship (dashed line) of single RyR2 WT channels was taken from a previous study [46] where E4146K was part of the study.

We next assessed whether the E4146K mutation alters the cytosolic Ca^2+^ activation of RyR2. We performed single channel analysis of the RyR2 WT and the E4146K mutant in the presence of various cytosolic Ca^2+^ concentrations (45 nM to 100 µM) and in the near absence of luminal Ca^2+^ (∼45 nM). As shown in Figure 7, single RyR2 WT channels were activated by cytosolic Ca^2+^ (∼100 nM), whereas, the E4146K mutation substantially reduced the cytosolic Ca^2+^ activation of RyR2 (Figure 7A,B). Taken together, these single-channel analyses indicate that RyR2 mutation E4146K suppresses both the cytosolic and luminal Ca^2+^ activation of RyR2.

### The LOF G4935R mutation exerts dominant negative impact on RyR2 WT function

**Figure 8:**
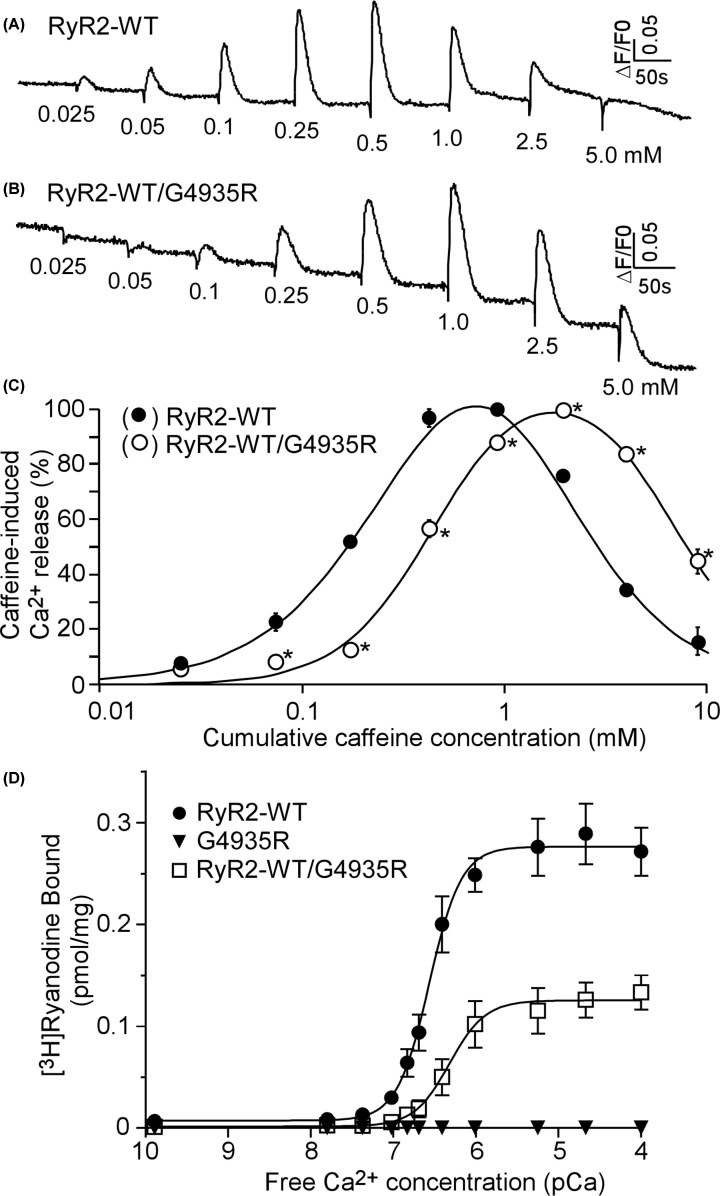
The RyR2 LOF G4935R mutation has dominant negative effect on RyR2 WT HEK293 cells were transfected with RyR2 WT (**A**) or co-transfected with RyR2 WT and G4935R (**B**). Fluorescence intensity of the fluo-3-loaded transfected cells was monitored continuously before and after each caffeine addition. (**C**) Cumulative caffeine concentration–Ca^2+^ release relationships in HEK293 cells transfected with RyR2 WT or co-transfected with RyR2 WT/G4935R. Data shown are mean ± SEM (*n*=3). (**D**) [^3^H]ryanodine binding to cell lysates prepared from HEK293 cells transfected with RyR2 WT or the G4935R mutant or co-transfected with RyR2 WT/G4935R was carried out at various Ca^2+^ concentrations (0.1 nM to 100 µM). Data shown are mean ± SEM (*n*=5 separate experiments for WT, 3 for RyR2 WT/G4935R, and 4 for G4935R. *P <0.05 vs.WT.).

The G4935R mutant does not form a caffeine-sensitive Ca^2+^ release channel in HEK293 cells when expressed alone. Besides abolishing its own channel function, the G4935R mutation may affect the function of the RyR2 WT channel when co-expressed with the WT. To test this possibility, we co-transfected HEK293 cells with RyR2 WT and the G4935R mutant plasmid cDNAs (in 1:1 ratio) and assessed the caffeine induced Ca^2+^ release in these co-transfected cells. As shown in Figure 8, co-expression of the G4935R mutant significantly suppressed the caffeine response of RyR2 WT (Figure 8A–C). We also assessed the impact of the G4935R mutation on [^3^H]ryanodine binding to RyR2. Similarly, we found that the G4935R mutation completely abolished Ca^2+^ dependent activation of [^3^H]ryanodine binding to RyR2 when it was expressed alone (Figure 8D). Furthermore, co-expression of the G4935R mutant with RyR2 WT substantially inhibited Ca^2+^ dependent activation of [^3^H]ryanodine binding to RyR2 (Figure 8D). Thus, the LOF G4935R mutation exerts a dominant negative effect on the function of the RyR2 WT channel.

## Discussion

It is generally believed that RyR2-associated ventricular arrhythmias (VAs) and SCD result from GOF defects of the RyR2 channel [2–5]. However, we and others have recently shown that a number of RyR2 mutations associated with IVF and SCD with negative exercise-stress testing suppress the function of RyR2 [8,46,55–58]. Since only a fraction of known disease-associated RyR2 mutations have been functionally characterized, it is possible that additional LOF RyR2 mutations may exist. To test this possibility, we assessed, in the present study, the functional impact of 18 RyR2 mutations that were labeled as IVF or associated with sudden death. We found two additional LOF RyR2 mutations (E4146K and G4935R). A hallmark of CPVT patients with GOF RyR2 mutations is reproducible exercise-induced bidirectional and/or polymorphic VAs [2–5]. Given their opposite impact on the RyR2 channel, LOF RyR2 mutations would expect to be associated with different clinical phenotypes in patients. Indeed, exercise stress testing (EST) showed no evidence of arrhythmias in the patient carrying the LOF G4935R mutation associated with SCD [64]. The LOF RyR2-E4146K mutation was discovered by postmortem genetic testing in a 14 years old male with a family history of sudden death who died during sleep [59]. Thus, consistent with those reported recently [46], LOF RyR2 mutations are associated with VAs that are distinct from the typical CPVT.

The existence of both GOF and LOF RyR2 mutations has profound implication for the understanding of RyR2-associated cardiac arrhythmias. The GOF RyR2 mutations are believed to enhance the propensity for spontaneous Ca^2+^ waves under conditions of SR Ca^2+^ overload such as during physical and emotional stress [5]. These spontaneous Ca^2+^ waves are well-known cause of DADs, which, in turn, can lead to triggered activity, triggered arrhythmia, and SCD [5,31–33]. However, the exact mechanism by which LOF RyR2 mutations causes sudden death has yet to be defined. We have recently shown that the RyR2 LOF mutation D4646A resulted in substantial electrophysiological and structural remodeling and enhanced the propensity for Ca^2+^ alternans, EADs, and re-entrant activities [46]. Therefore, there are at least two distinct mechanisms by which RyR2 mutations could lead to VAs: a DAD-based mechanism associated with enhanced RyR2 function and an EAD-mediated re-entrant mechanism associated with suppressed RyR2 function.

Given these fundamental differences in their functional impact, the diagnosis and treatment strategies for ventricular arrhythmias associated with GOF or LOF RyR2 mutations would have to be different. EST is widely used for the diagnosis of typical CPVT, which is associated with GOF RyR2 mutations. However, such a test would be ineffective in identifying patients carrying LOF RyR2 mutations because of their lack of response to exercise stress test [46]. In this regard, it is of interest to know that the LOF RyR2 G4935R mutation identified in an 8 years old girl who suffered from SUD was initially given a clinical diagnosis of epilepsy [64]. Hence, a specific diagnostic test for individuals with RyR2 LOF mutations is pressingly needed. We have recently established a programed electrical stimulation sequence that encompasses a long burst, a long pause, and a short-coupled extra-stimulus (LBLPS). We showed that this LBLPS stimulation sequence triggers VAs in RyR2 LOF mutant mice, but not in RyR2 GOF mutant mice or WT control mice. However, the specificity and sensitivity of the LBLPS protocol in humans remain to be determined. From a clinical standpoint, there are currently no studies directly comparing the response to therapies of GOF vs LOF RyR2 mutant carriers. Drugs that suppress RyR2 activity would be appropriate for treating patients with enhanced RyR2 function. However, these drugs would be ineffective for patients with suppressed RyR2 function as they would exacerbate the LOF defects. For an effective treatment, one would need to suppress the GOF of RyR2, whereas reverse the LOF of RyR2. It is, therefore, necessary and important to characterize the functional impact of all disease-associated RyR2 mutations, so that one could distinguish patients with enhanced RyR2 function from those with suppressed RyR2 function for a proper diagnosis and treatment of RyR2-assoicated cardiac arrhythmias.

It is of interest to note that although mutations G4935R, S4938F, and R4959Q are all located in the CTD, their impact on RyR2 channel activation is very different. R4959Q does not significantly alter caffeine activation of RyR2, while S4938F markedly suppresses caffeine activation, and G4935R completely abolishes caffeine activation. Thus, it would be challenging to predict whether a mutation will impact channel function solely based on the domain it is located in.

Another interesting observation is that the presence of caffeine (10 mM), but not the absence of caffeine, induced Ca^2+^ oscillations in RyR2-E4146K mutant expressing HEK293 cells. On the other hand, high concentrations of caffeine induced a single peak of Ca^2+^ release in RyR2-WT expressing HEK293 cells. The exact mechanism underlying this difference is not completely understood. We have shown previously that caffeine induces Ca^2+^ release/Ca^2+^ oscillations in RyR2-expressing HEK293 cells by reducing the threshold for SOICR [66]. The observation that SOICR in RyR2-E4146K expressing HEK293 cells occurs only in the presence, but not in the absence of high concentrations of caffeine is consistent with the notion that the threshold for SOICR in RyR2-E4146K expressing HEK293 cells is markedly increased. We hypothesize that this markedly elevated SOIR threshold prevents store overload induced spontaneous Ca^2+^ oscillations in the absence of caffeine. Whereas, in the presence of 10 mM caffeine, the SOICR threshold in RyR2-E4146K expressing HEK293 cells would be reduced to a level at which spontaneous Ca^2+^ oscillations can now occur. On the other hand, the normal SOICR threshold in RyR2-WT expressing HEK293 cells would be dramatically reduced by 10 mM caffeine, resulting in the depletion of ER Ca^2+^ stores, which may explain the lack of Ca^2+^ oscillations in RyR2-WT expressing HEK293 cells in the presence of 10 mM caffeine [66].

In summary, the present study reveals novel LOF RyR2 mutations associated with IVF and SCD with negative exercise stress test. IVF and SCD associated with loss of RyR2 function represents a new entity of ventricular arrhythmias distinct from CPVT. Different strategies and protocols will be required for the diagnosis and treatment of ventricular arrhythmias associated with enhanced or suppressed RyR2 function.

## Supplementary Material

Supplementary Figures S1 and S2 and Supplementary Data

## Data Availability

All data are available in the main text or the supplementary information.

## Abbreviations

AF: atrial fibrillation
BSA: bovine serum albumin
CPVT: catecholaminergic polymorphic ventricular tachycardia
CRDS: Ca^2+^ release deficiency syndrome
DAD: delayed afterdepolarization
DMEM: Dulbecco’s Modified Eagle’s Medium
EAD: early-afterdepolarization
EST: exercise stress testing
Fura-2 AM: Fura-2 acetoxymethyl ester
GOF: gain-of-function
HEK293: human embryonic kidney 293
IVF: idiopathic ventricular fibrillation
KRH: Krebs–Ringer–HEPES
LOF: loss-of-function
PBS: phosphate-buffered saline
RyR2: cardiac ryanodine receptor
SCD: sudden cardiac death
SOICR: store overload induced Ca^2+^ release
SR: sarcoplasmic reticulum
SUD: sudden unexplained death
WT: wildtype
