# Supplementary material for: Identification of loss-of-function RyR2 mutations associated with idiopathic ventricular fibrillation and sudden death
Source: Biosci Rep. 2021 Apr 22;41(4):BSR20210209. doi: 10.1042/BSR20210209 (PMC8062958; doi:10.1042/BSR20210209)
Supplement: Supplementary Figures S1 and S2 and Supplementary Data [file BSR-2021-0209_supp.pdf]

Supplementary Figure 1

Uncropped Western blots for Figure 2E

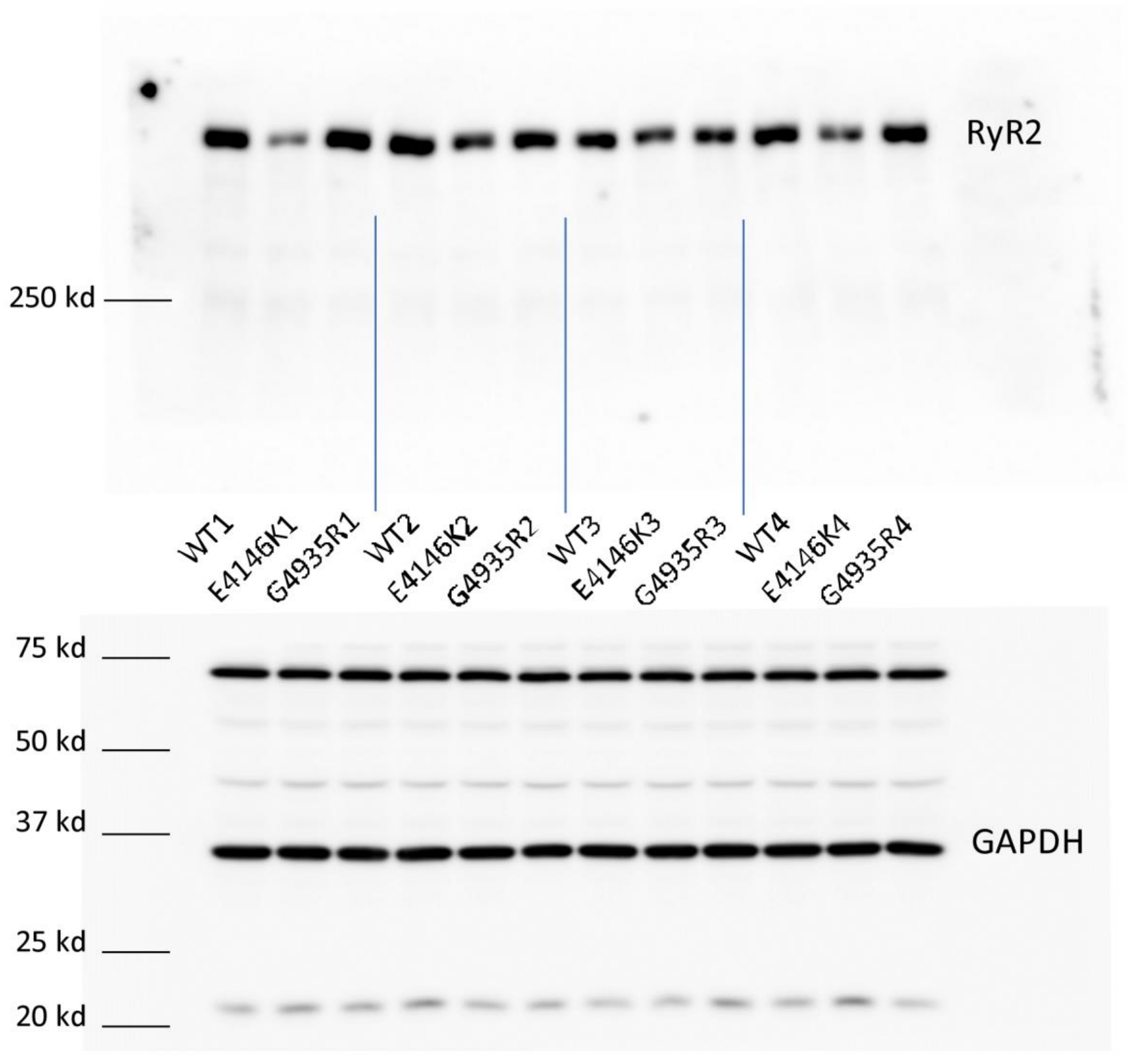

Supplementary Fig. 1 Effect of IVF-associated RyR2 mutations on channel expression
HEK293 cells were transfected with RyR2 WT, E4146K, or G4935R. Cell lysates were prepared from these transfected cells and used for immunoblotting analysis. The same amount of cell lysate was used for immunoblotting using the anti-RyR2 antibody.

Supplementary Figure 2

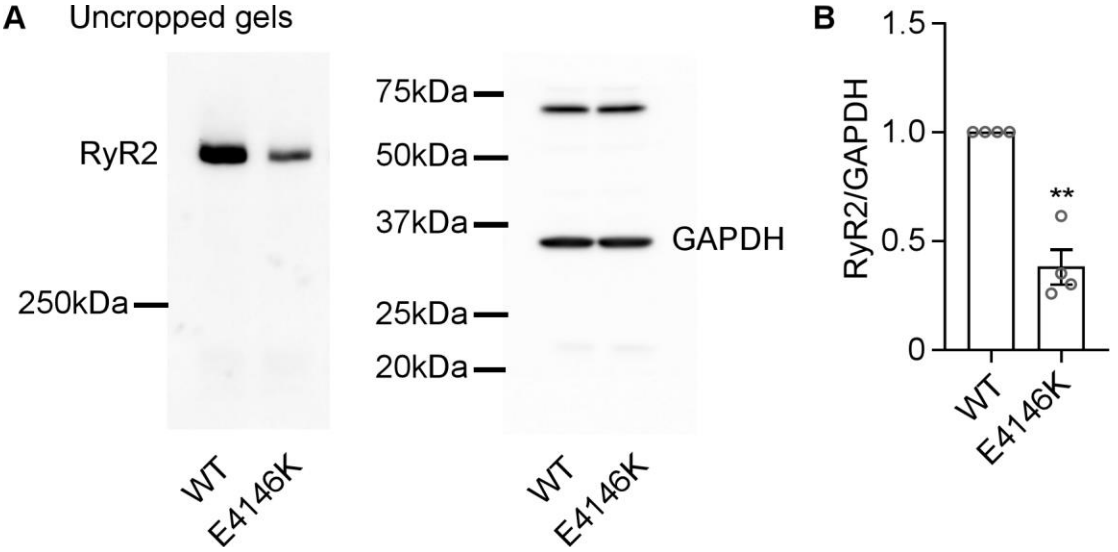

Supplementary Fig. 2 Effect of E4146K mutation on channel expression in HEK293 cells

Cell lysates were prepared from stable, inducible HEK293 cells expressing RyR2 WT or the E4146K mutant and used for immunoblotting analysis (**A, B**). The same amount of cell lysate was used for immunoblotting using the anti-RyR2 antibody. Uncropped gels for anti-RyR2 and anti-GAPDH immunoblots are shown (**A**). Data shown are mean ± SEM (n=4) (**B**).

Supplementary Information

Of the 18 RyR2 mutations tested in the present study, three are novel and the rest have been reported previously. A detailed description of the clinical evaluation for each of the three novel RyR2 mutations (E1127G, A3442E, and I3476T) was provided below. A brief summary of the major phenotypes for each of the previously reported RyR2 mutations was also included. Please refer to the published literature for more detailed description of the previously reported RyR2 mutations [6, 11, 13, 59-65].

I217V

The RyR2-I217V mutation was reported by Tester et al. [59] through molecular autopsy in a 21-year-old male who died suddenly and had syncope and a positive family history of SCD, but the SUD event was nonspecific [59].

R414C

The RyR2-R414C mutation was reported by Creighton et al. [60] through molecular autopsy in a 16-year-old female who died suddenly while swimming competitively. The patient had a history of attention deficit hyperactivity disorder and syncope. Evaluations with electroencephalogram, computed tomography scan, and 24-hour Holter monitoring were negative. Histological analyses showed unremarkable myocardium, coronary arteries and conduction system. Exercise stress test was not available [60].

P446A

The RyR2-P446A mutation was reported by Tester et al. [61] in a 9-year-old boy who had ventricular fibrillation when he was 11-month-old and a family history of sudden infant death syndrome (SIDS) and SCD. Exercise stress test was not available. The patient had syncope during breath holding and aborted cardiac arrest [61].

E1127G

The RyR2-E1127G mutation has not been reported previously. The patient is a young, completely asymptomatic female who experienced an aborted sudden cardiac death (April 2011) at 25 years of age. At the moment of the event the patient was resting waiting for the start of a theatre act where she was supposed to sing. She experienced a sudden loss of consciousness and ventricular fibrillation was observed on the cardiac monitor by paramedics who were on place; sinus rhythm was promptly restored with DC shock. The subsequent clinical and instrumental investigations showed normal findings (ECG, echocardiography, coronary angiography and heart MRI). Exercise stress test and ECG Holter monitoring did not show any arrhythmias. Therefore, on May 2012 the patients underwent ICD implantation and was referred to our center for genetic investigations. Genetic screening of KCNQ1, KCNH2, SCN5A, KiR2.1, KCNE1 and KCNE2 were negative while the screening of RyR2 led to the identification of the E1127G mutation. Collection of family history showed a case of sudden death in a young male who died suddenly at rest at the age of 13. Unfortunately, no DNA was available for cascade genetic screening in the family. The E1127G mutation was inherited from the asymptomatic father who presented with a normal ECG (but refused additional clinical investigations). The patient was treated with beta-blockers and remained asymptomatic thereafter with no arrhythmias detected by the device.

G2145R

The RyR2-G2145R mutation was reported by Marjamaa et al. [62] in the index patient who was 41 years old at the time of death. The patient had a syncopal event at exercise two months before his death. A medico-legal autopsy and histological and toxicological analyses revealed a structurally normal heart and provided no apparent explanation for the sudden death. The index patient's daughter is also RyR2 G2145R mutation positive, but showed no structural or electrical abnormalities on clinical evaluation at the age of 23 years old [62].

F2331S
The RyR2-F2331S mutation was reported by Creighton et al. [60] through molecular autopsy in an 8-year-old male who became unresponsive while climbing a rock wall. The individual had prior seizure episodes. Previous clinical evaluation did not show any evidence of long QT syndrome. Histological analyses at autopsy showed unremarkable myocardium, coronary arteries and conduction system [60].

G2337V
The RyR2-G2337V mutation was reported by Haugaa et al. [63] through molecular genetic screening for CPVT and long QT syndrome in a family with eight members who died suddenly. Post-mortem genetic testing in three of them revealed heterozygous RyR2 G2337V mutation. All patients had normal resting ECG and none of the subjects experienced sustained ventricular tachycardias or required external cardioversion [63].

A2387T
The RyR2-A2387T mutation was reported by Tester et al. [61] in an 18-year-old female who experienced aborted cardiac arrest (ACA) and had a positive family history of SCD. Exercise stress testing was not available [61].

Y2392C
The RyR2-Y2392C mutation was reported by Bauce et al.[13] through screening families with effort-induced polymorphic ventricular arrhythmias, syncope, and sudden death. The 12-lead ECG showed normal sinus rhythm and atrioventricular conduction and postmortem examination showed structurally normal heart [13].

R2401L
The RyR2-R2401L mutation was reported by Creighton et al.[60] through molecular autopsy in a 12-year-old male who died from a syncopal/arrhythmic episode while running. The patient had a history of exercise-induced ventricular tachycardia and sinus node dysfunction. A previous catheterization study showed normal hemodynamics. Histological analyses showed unremarkable myocardium, coronary arteries and conduction system [60].

A3442E
The RyR2-A3442E mutation has not been reported previously. The proband is a previously asymptomatic female patient who presented with syncopal episode with detection of ventricular fibrillation treated with DC Shock and external cardiac massage. The event was triggered by acute emotion. At the moment of the event (April 2002) the patient was 16 years old. Since then, she performed several tests including MRI and electrophysiological study with flecainide with negative results. Ventricular extrasystoles (couplets and bidirectional triplets) during exercise testing were observed. After about a month, new pre-syncopal event after an intense emotion

occurred. During the visit in August 2002 at our center, the presence of exercise-induced PVCs was confirmed. On this base, even if there was no clear evidence of repetitive life-threatening arrhythmias we decided to start with beta-blocker therapy (nadolol 40 mg/day approx 1 mg/kg). Another exercise stress test on therapy showed no arrhythmias. However, in agreement with the family an ICD was implanted (October 2002). The device was replaced in March 2010 (including ICD lead). During the visit (November 2013) the patient was regularly taking 40 mg/day of nadolol, no arrhythmias were detected at device interrogation. Exercise stress test showed ventricular bigeminy inducible with FC >120 bpm (reached at high workload after 10 minutes of exercise at Bruce IV), echocardiogram was normal; Holter monitoring showed isolated PVCs and one couplet during HR increases. Genetic screening resulted positive for the presence of a RyR2 A3442E mutation. Both parents were negative (sporadic case).

I3476T

The RyR2-I3476T mutation has not been reported previously. The proband is a female subject born on March 1989 who was referred for cardiologic evaluation for recurrent syncopal spells starting at the age of 2 years usually triggered by acute emotional stress. In 1995 while the patient was rushed to the ER due to a new episode of loss of consciousness a polymorphic VT was recorded (in the available medical record the arrhythmias was referred as “torsade de pointes-like”). This evidence in combination with a QTc at the upper limit immediately after restoration of sinus rhythm led the local referring cardiologist to suspect the diagnosis of LQTS and beta-blocker therapy was undertaken. The patient was referred to our center for clinical and genetic evaluation. The initial evaluation of the available ECGs showed normal QTc. We performed cardiac echo (normal), additional ECGs (unremarkable) and 12-leads Holter monitoring (normal QT no arrhythmias). However, polymorphic ventricular couplets and triplets were reproducibly induced at the exercise stress test. For these reasons we carried out molecular analysis of cardiac ryanodine receptor that led to the identification of the I3476T mutation. The analysis of the major LQTS genes was negative. Cascade genetic testing in the family led to the identification of the same genetic defect in the proband’s father (asymptomatic, with normal cardiac findings including exercise stress test).

R3570W

The RyR2-R3570W mutation was reported by Marjamaa et al. [62] independently in two victims of SCD from Eastern Finland. The index patient in one family died at the age of 17 years old during a volleyball game. An autopsy and toxicological examination revealed a moderately enlarged and dilated heart. The index patient in another family was a 55-year-old male died suddenly while carrying a water bucket. Postmortem investigations uncovered a moderately enlarged and dilated heart. In these two families, 20 individuals out of 64 family members screened were carriers of the RyR2 R3570W mutation. None of the relatives was reported to have syncopal events or palpitations [62].

N4097S

The RyR2-N4097S mutation was discovered by molecular autopsy in an 18-year-old male who died suddenly and had a positive family history of SCD, but the SUD event was nonspecific [59].

E4146K

The RyR2-E4146K heterozygous mutation was reported by Tester et al.[59] through molecular autopsy in an 14-year-old male who died suddenly during sleep and had a positive family history of SCD [59].

I4848V

The RyR2-I4848V mutation was reported by Tester et al. [61] in two females (14 and 35 years old) who had near-drowning experiences and a positive family history of SCD. One patient also had aborted cardiac arrest during exertion [61].

G4935R

The RyR2-G4935R heterozygous mutation was reported by Johnson et al. [64] in a female patient presented with episodes of abrupt loss of consciousness while running at the age of 4 years old. The patient had a prenatal history of bradycardia in utero with no identified etiology. There was family history of seizures, but no history of sudden unexplained death. Neurologic examination was normal. Multiple ECGs were performed, all of which were normal. Exercise stress testing also showed no evidence of arrhythmia. At 8 years of age while running, the patient abruptly fell to the ground and had a prolonged generalized tonic-clonic seizure and died from cardiopulmonary arrest [64].

R4959Q

The RyR2-R4959R mutation was reported by Laitinen et al. [65] in a 60-year-old female proband during screening for RyR2 mutations in CPVT families. The proband had experienced syncope at the age of 40 years old. Resting ECG was normal, and cardiac imaging studies did not demonstrate any evidence of cardiac structural abnormalities [65].The same mutation was also reported by Tester et al. [61] in a 31-year-old female and a 12-year-old female who presented with seizure and syncope during exertion and had a family history of sudden cardiac death. Exercise stress testing showed PVCs, ventricular bigeminy, and couplets.
